# Significance of *Demodex folliculorum* and *Demodex brevis* in Pathogenesis of Dermatological Diseases—Current State of Knowledge

**DOI:** 10.3390/medicina61040660

**Published:** 2025-04-03

**Authors:** Katarzyna Rychlik, Julia Sternicka, Roman J. Nowicki, Leszek Bieniaszewski, Dorota Purzycka-Bohdan

**Affiliations:** 1 Department of Dermatology, Venereology and Allergology, Medical University of Gdańsk, University Clinical Centre, 80-214 Gdańsk, Polandroman.nowicki@gumed.edu.pl (R.J.N.); 2 Mycology Outpatient Clinic, University Clinical Centre, 80-214 Gdańsk, Poland; 3 Clinical Physiology Unit, Medical Simulation Centre, Medical University of Gdańsk, 80-204 Gdańsk, Poland; leszek.bieniaszewski@gumed.edu.pl

**Keywords:** *Demodex folliculorum*, *Demodex brevis*, demodicosis, rosacea, blepharitis

## Abstract

*Demodex folliculorum* and *Demodex brevis* are external parasites that reside in human hair follicles and sebaceous glands, most commonly on the cheeks, chin, nose, and eyelids, inhabiting the eyelash follicles. The prevalence of *Demodex* spp. varies with age. The highest concentration of mites is observed in older people, being almost 100%, and the lowest is found in children. Although the presence of parasites does not directly lead to the development of pathological symptoms, their high density is associated with diseases such as rosacea or blepharitis. This manuscript delves into the biological characteristics of *Demodex folliculorum* and *Demodex brevis* with consideration of current diagnostic techniques for detecting *Demodex* mites. It also aims to provide an in-depth analysis of the role *Demodex* mites play in the development of various dermatological conditions, with a review of the current therapeutic approaches for managing *Demodex*-related diseases.

## 1. Introduction

*Demodex folliculorum*, which is typically found in hair follicles, and *Demodex brevis*, residing in sebaceous glands and Meibomian glands, are two species of *Demodex* that can occur in humans [1]. They are both common commensals occupying pilosebaceous areas, mainly inhabiting the face, scalp, and upper chest. *Demodex* mites, together with diverse communities of microorganisms, including bacteria, viruses, and fungi, form the skin microbiome [2]. It follows that those parasites can be present in people without inducing any symptoms. It has been found that the prevalence of these mites in people over 60 years of age is about 84%, and almost 100% in those over 70 years of age [3]. However, when present in excessive amounts, mites can play a pathogenic role [4]. The presence of mites numbering above five per 1 cm^2^ of skin can be associated with the appearance of skin lesions. The possible role of *Demodex folliculorum* in the development of rosacea, perioral dermatitis, blepharitis, folliculitis, and pityriasis folliculorum is emphasized [5]. The aim of this work is to summarize the current state of knowledge on characteristics and role of *Demodex* spp. in human health. To achieve that, a literature review was conducted using PubMed. The following terms were used for the search: “*Demodex*”, “*Demodex brevis*”, “*Demodex folliculorum*”, “demodicosis”, “acne rosacea”, “blepharitis”. Articles in Polish and/or English containing the above-mentioned key words were analyzed.

## 2. Characteristics of *Demodex* spp.

*Demodex* mites are ectoparasites that belong to the Demodicidae family of the order Acari from the class Arachnida. *Demodex folliculorum* and *Demodex brevis* are two species of mites that occur in humans. *D. folliculorum* is larger, measuring around 0.3–0.4 mm in length, whereas *Demodex brevis* is smaller, typically about 0.2–0.3 mm long [3]. *D. folliculorum* is more common than *Demodex brevis* and usually resides in follicle funnels. *Demodex brevis* penetrates deeper, into the sebaceous ducts and Meibomian glands, and has spindle-shaped egg cells (60 μm × 34 μm) [6]. The life cycle of mites lasts from 14 to 18 days, with development occurring in a single host. *Demodex* spp. go through five development stages: eggs, larval forms, protonymphs, nymphs, and adults. Female mites are more rounded and larger than males. Mating occurs within the hair follicle, where the females lay eggs. After 3–4 days, six-legged larvae hatch and, in approximately 7 days, mature into adults [7]. *Demodex* feeds on the sebum and cellular proteins obtained by a protease-containing salivary enzyme [8]. This is why *Demodex* mites are most commonly found in areas with a high concentration of sebaceous glands, such as the nose, chin, cheeks, and around the eyes. *Demodex folliculorum* specifically resides in the eyelash follicles, using its claws to scrape the inner walls of the eyelashes, causing the follicles to dilate, leading to epithelial hyperplasia and reactive hyperkeratinization. This suggests the formation of so-called cylindrical dandruff (CD), which is considered to be a pathognomonic symptom indicating *Demodex folliculorum* invasion of eyelash follicles. *Demodex* mites can be transmitted from one person to another through direct skin contact. Additionally, makeup products shared by different individuals within short periods (ranging from a few hours to several days) can also serve as a source of transmission. *Demodex* spp. has been found in all human races of adult populations, but rarely in newborns and children [9]. The number of mites has been proven to increase with age, being almost 100% among elderly. A study conducted in patients from northern Poland with suspicion of demodicosis showed that in the analyzed population of patients, the likelihood of detecting *Demodex* spp. was positively correlated with the patient’s age, with the mites primarily found on the eyelid margins and facial skin. Furthermore, men were more likely than women to harbor *Demodex* [10]. It has also been found that *Demodex* mites are more prevalent in obese patients, those with high blood sugar levels, those in the end-stage of chronic kidney disease, and those with insufficient or reduced immunity [11]. The diagnosis of demodicosis relies on correlating clinical symptoms with the findings from diagnostic tests, such as direct microscopic examination (DME), standardized skin surface biopsy (SSSB), or different, more novel techniques. In both of these methods, collected samples are evaluated under a light microscope; however, the way the material is obtained differs between the methods. Standardized skin surface biopsy is the most commonly applied method. The process starts by drawing a 1 cm^2^ square on one side of the slide. Then, cyanoacrylic glue is applied to the opposite side of the slide, which is subsequently pressed against the skin lesions. After allowing the glue to dry, the slide is removed, one drop of oil is added, and everything is covered with a cover slip. The samples collected can be evaluated under a light microscope at lower magnification (×20–40) to visualize the *Demodex* mites [12]. DME involves selecting a 1 cm^2^ area of pathologically changed skin and using a comedone extractor to squeeze it. The resulting sample is then placed in a drop of 10% potassium hydroxide and covered with a cover slip. The collected samples can also be examined under a light microscope at the same magnification as in SSSB [11]. To confirm demodicosis, a *Demodex* density above five per 1/cm^2^ is required [13]. A study by Ü. Aşkın, D. Seçkin shows that SSSB exhibits higher sensitivity compared to DME [12]. However, Yun et al. came to the opposite conclusion, showing in their study that DME is characterized by greater accuracy than SSSB [14]. DME does not display hair follicles, which are crucial for detecting *Demodex* spp. [12]. On the other hand, SSSB has limitations related to the small area of the examined surface, the lower depth of the collected material, and the poor adhesion of mites to the microscope slide, which may result in false negative results [13,14]. DME detects both *Demodex folliculorum* and *Demodex brevis*, while SSSB detects only *Demodex brevis* [4]. DME is a simple, effective, and time-saving test for detecting mites and is comparable to SSSB [15]. Modern techniques for identifying *Demodex* spp. involve confocal microscopy (RCM) and high-definition optical coherence tomography (HD-OCT). These are fast, visual techniques, but due to low availability and high costs, they have limited applications [5].

### 2.1. Dermatological Diseases Associated with Demodex spp. Invasion

**Figure 1:**
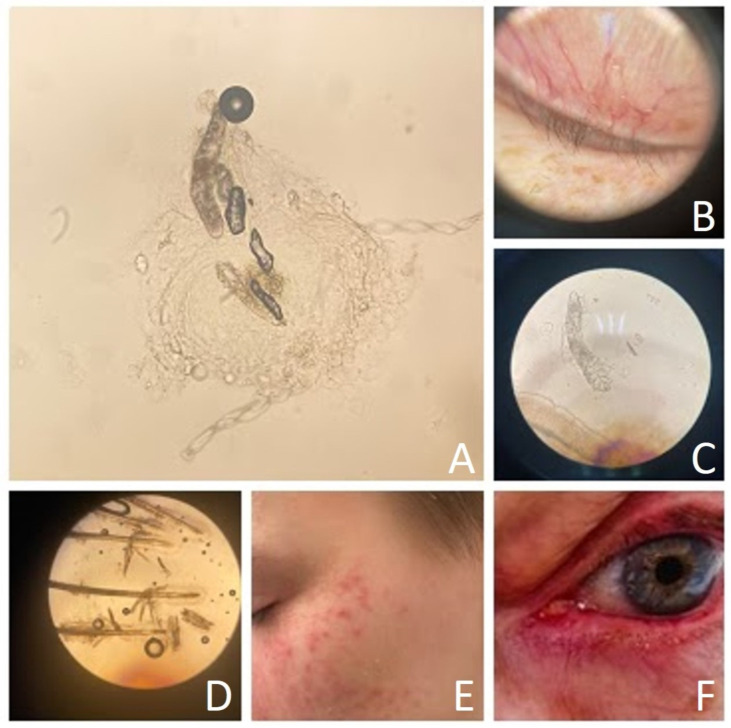
*Demodex* mite and demodex-related diseases ((**A**)—*Demodex folliculorum*, light microscope magnification 10 × 10; (**B**)—eyelid of patient with blepharitis, dermatoscopy; (**C**)—*Demodex brevis*, light microscope magnification 10 × 40; (**D**)—*Demodex folliculorum* and *Demodex brevis*, light microscope magnification 10 × 10; (**E**)—patient with erythema, papules, and pustules, (**F**)—patient with erythema of upper and lower eyelid).

The exact role of *Demodex* spp. in dermatological conditions is not yet fully understood. Low-density mites are commonly found in the pilosebaceous follicles of adults and are considered normal. However, an overgrowth of these mites, accompanied by visible whitish scales at the hair follicle base and symptoms such as erythema, burning, itching, hypersensitivity, or skin roughness, is typically referred to as demodicosis. “Demodicosis” or “demodicidosis” describe skin conditions caused by *Demodex* mites (Figure 1). These conditions are classified into primary, such as pityriasis folliculorum, and secondary, which include rosacea, acne vulgaris, perioral dermatitis, and blepharitis [16]. Anastasia Kargadouri et al. examined the prevalence of demodicosis in patients with specific clinical symptoms who visited the outpatient clinic of Andreas Syggros Dermatological Hospital in Greece. In this retrospective study, an association between manifestations such as itching, cheek redness, skin sensitivity, and the presence of demodicosis have been observed [17]. Ezgi Aktaş Karabay et al. [8] showed in their study that *Demodex* invasion was associated with acne vulgaris (AV), rosacea, and seborrheic dermatitis (SD). The prevalence of *Demodex* was significantly higher in patients with rosacea than in those with AV, those with SD, and the control group [8].

Rosacea is a prevalent chronic inflammatory condition that affects 1% to 10% of the global population. The primary clinical symptoms include persistent redness, telangiectasia, and the presence of papules and pustules on the central areas of the face [18]. The development of rosacea is linked to several exacerbating or triggering factors, such as emotional stress, microbial invasion, changes in temperature, sun exposure, physical activity, consumption of hot beverages and spicy foods, and exposure to airborne pollen [19]. Patients with rosacea also tend to have ocular symptoms, including dryness, photophobia, conjunctivitis, and blepharitis. Given that rosacea is located on the face, it negatively affects quality of life and comfort of the patients [20].

Rosacea is typically regarded as a disorder of both innate and adaptive immunity, which can be triggered by various factors. Some experts argue that these immune responses are secondary, resulting from an abnormal increase in *Demodex* populations, likely induced by local immunosuppressive factors, hypervascularization, or sebaceous gland hyperplasia. Mites may also promote vasodilation, creating a vicious cycle where hypervascularization encourages further *Demodex* proliferation, which in turn triggers an inflammatory response leading to additional vasodilation. Additionally, there may be another cycle at play: immunosuppression can enhance the growth of parasites, which likely secrete local immunosuppressive factors that aid their survival [21]. Gonzalez-Hinojosa et al. highlighted a distinct predominance of mites in patients with rosacea, offering new insights into the potential link between external factors and the immune response in the pathophysiology of this condition [22]. It has been demonstrated that bacterial antigens found in *Demodex* mites can trigger lymphocyte proliferation. Given that lymphocyte activation is more common in patients with rosacea than in healthy individuals, there may be an inherent susceptibility to the pathogenic effects of the mites [23,24]. Lacey et al. observed that high concentrations of *Demodex* mites can provoke an immune response by activating the Toll-like receptor 2 pathway, suggesting a potential role in the development of rosacea [25]. The study “Erythematotelangiectatic rosacea may be associated with a subclinical stage of demodicosis” by F. Forton and V. De Maertelaer supports this connection. The increased expression of type 2 Toll-like receptors (TLR2), cathelicidin, IL-37, and leukocyte activity in rosacea further suggests that microorganisms contribute to its pathogenesis. Mites carry antigens that can stimulate the host’s adaptive immune system, including mucopolysaccharidic endocuticle, exocuticle protein, endobacteria, and proteases [24,25,26]. In patients affected by papulopustular rosacea, positive tests for *Demodex* spp. are more often observed compared to other patients. *Demodex folliculorum* is found more frequently on the faces and scalps of patients with rosacea compared to control groups, although there is no statistical correlation with scalp symptoms. The scalp may serve as a reservoir for *Demodex* mites, which could potentially migrate back to the face after mite-targeting therapy [27]. On the other hand, a meta-analysis of the frequency and degree of *Demodex* mite invasion in individuals with rosacea, conducted by Yin-Shuo Chang et al., showed that both erythematotelangiectatic rosacea and papulopustular rosacea were characterized by significantly higher *Demodex* density than that found in control patients [18]. It was also established that rosacea is a significant risk factor for Demodex infestation in the eyelashes, independent of age and gender, and occurs more frequently in the papulopustular form. The higher prevalence of Demodex in the hair follicles of rosacea patients could have therapeutic implications, potentially leading to symptom alleviation and modification of the disease course [22]. A study from the University of Turkey revealed that the Demodex burden in rosacea patients was approximately 30 times higher than in healthy controls. The study also suggested that mite infestation, particularly co-infection with *Demodex folliculorum* and *Demodex brevis*, may act as a potential trigger for rosacea, which should not be overlooked in clinical practice, and that it may be beneficial to initiate antiparasitic treatment upon detecting the parasite [28]. Ilaria Trave et al. explored potential correlations between the residence of *Demodex* mites and clinical features, finding that patients with rosacea had a higher frequency of positive results in both microscopic examination and PCR tests compared to controls. Those who received a positive result for samples taken from their facial skin also received more frequent positive results for samples from their scalp. This area may constitute a reservoir of *Demodex* mites and should be examined using sensitive and painless methods, e.g., the PCR method [29]. Skin changes caused by obesity may predispose patients with rosacea to *Demodex* infection. Comparing obese and non-obese patients, it was noticed that obese patients had significantly higher rates of erythematotelangiectatic rosacea, rosacea severity, and redness-related complaints. Moreover, the results showed that *Demodex* infection was significantly more common in obese patients, which leads to the conclusion that these dependencies may be related [30].

Acne vulgaris is a multifactorial condition affecting the pilosebaceous unit, commonly seen in adolescents and young adults, yet not limited to those age groups. Key pathophysiological factors include hyperseborrhea, altered keratinization, Cutibacterium acnes infection, and inflammation. *Demodex* mites can induce perifollicular inflammation through their protease and lipase enzymes, leading to follicular occlusion and the release of inflammatory cytokines. In studies by Arzu Ferhatosmanoğlu, it was found that the prevalence of *Demodex* in acne patients was 26.6%, comparable to that of healthy individuals, with no correlation observed between acne severity and *Demodex* presence [31]. On the other hand, in a meta-analysis conducted by Ya-e Zhao et al., it has been proven that acne vulgaris is associated with *Demodex* invasion [32]. However, the role of mites in acne vulgaris remains a topic of debate. While several studies indicate a positive correlation between acne vulgaris and Demodex infestation, other research challenges this connection [1]. High prevalence and density of parasites is associated with acne accompanied by nonspecific facial dermatitis, but no such correlation is certain for acne vulgaris alone. Although previous reports have indicated a higher prevalence of Demodex in patients with discussed dermatosis, this does not confirm that the mites cause this disease. The reasons for their increased presence in these patients remain unclear. One possible explanation is that *Demodex* mites and acne vulgaris may be causally related through various mechanisms [1]. These may include, e.g., mechanical blockage of follicles by mites, leading to distension and intra-follicular hyperkeratosis. Their hard exoskeleton can act as a foreign body, potentially causing granuloma formation. When these mites breach the epithelial barrier, their antigens may trigger a type IV hypersensitivity reaction in the host’s immune system. Additionally, waste products from parasites and associated bacteria may trigger the innate immune system or activate delayed hypersensitivity reaction, which could be involved in acne development [33].

*Demodex* is commonly found in older individuals with dry eye syndrome. Itching and the presence of cylindrical dandruff were the most distinctive symptoms associated with *Demodex*. Orla Murphy et al., in a study conducted on the Irish population, proved that there is a solid correlation between the occurrence of *Demodex* and eye itching [34]. Ocular demodicosis is highly age-dependent and more common in older people. In a study conducted in a hospital in Poland, the prevalence of demodicosis was 77% in people over 70 years of age and 8% in people 25 years of age and younger. Several risk factors have been linked to ocular demodicosis, such as smoking, obesity, cancer, diabetes, and acquired immunodeficiency syndrome. A shared characteristic of these factors is their association with compromised immunity [35].

Eyelid and facial Demodex infection can coexist, as the presence of secondary blepharitis due to Demodex is linked to the increased density of mites on the face [36]. Blepharitis is an inflammatory condition affecting the eyes, eyelashes, and eyelids. It typically appears bilaterally and symmetrically. Demodex blepharitis can worsen vision quality and life quality. The symptoms significantly impact patients, leading to frequent visits to specialists and numerous unsuccessful attempts to find relief. The current treatment options for Demodex-induced blepharitis are cumbersome and may be ineffective and/or toxic to the eye [37]. People of any age, gender, and race can be infected with Demodex-induced blepharitis. Multiple studies have consistently demonstrated that the prevalence and impact of *Demodex* mites rise with age. It is believed that older patients, usually between 50 and 70 years old, have a weaker immune system and greater overall skin fragility, allowing mites to penetrate. Parasites may reproduce faster in older people due to increased skin pH and decreased skin hydration compared to younger patients [38]. Leslie O’Dell et al. conducted a multicenter study to assess the effect of Demodex blepharitis on patients’ quality of life and daily activities. Over half of the patients had experienced blepharitis symptoms for ≥4 years. Patients most commonly reported dry eyes, itching, and irritation, and 58% stated that they had never been diagnosed with blepharitis before. The researchers demonstrated that Demodex blepharitis has a significant negative impact on the mental and physical well-being and daily activities of affected patients [39]. In their study, Serife Akkucuk et al. proved a significant association between *Demodex* spp. invasion and chronic blepharitis and chalazion. The prevalence of parasites was notably higher in the blepharitis (75.5%) and chalazion (70%) groups compared to the control group (16.2%) [*p* < 0.001]. It was observed that mite presence increases with age in the blepharitis patient group [40]. Demodex-induced blepharitis is linked to a higher mite density on the face, confirming that eyelid and facial Demodex invasion can coexist, and a collaborative approach to treatment between eye care specialists and dermatologists should be encouraged [36]. This is especially important, because treating blepharitis is straightforward in its early stages, but chronic inflammation becomes challenging to manage [41].

### 2.2. Treatment of Demodicosis

Once diagnosed, treatment is initiated to decrease the number of *Demodex* spp. on the patient’s skin and alleviate clinical symptoms. The most commonly used topical treatments include 1% ivermectin, metronidazole, permethrin, and benzyl benzoate (Table 1).

Ivermectin is an anti-parasitic drug that can be implemented against a vast range of arachnids. It is rapidly absorbed and metabolized in the liver, reaching peak blood levels after 5 h. It can be administered orally or topically, boasting a good safety profile. When used systemically, the drug remains in the body for 36 h and is mainly excreted in feces (98%) and urine (1%). It selectively targets parasites by binding to glutamate-gated chloride ion channels in the peripheral nervous systems of invertebrates [42]. This interaction increases chloride ion permeability, leading to the paralysis and death of parasites and mites [43]. A *Demodex* mite die-off reaction may occur after oral ivermectin treatment, particularly in individuals with high mite density. Symptoms may temporarily get worse during the first few weeks before showing signs of improvement as therapy continues [44]. In a study conducted by Ilaria Trave et al., it was found that the overall success rate when treating *Demodex* infestation with ivermectin was 87.5%, with a relapse rate of 12.5%. Different studies have concluded that use of ivermectin results in a full response in 70% of patients. The median time before disease recurrence was 140 days, with an average time of 152 days [45]. Hui-Peng Huang et al. demonstrated in their study that topical ivermectin showed some effectiveness in mild cases of erythema-telangiectatic rosacea (ETR), although *Demodex* density was not assessed. Its erythema-reducing effects may result from its anti-inflammatory properties and the reduction of *Demodex*, which is a key activator of Toll-like receptor 2. Previous research has also shown that both topical ivermectin and a combination of ivermectin and carvedilol significantly improved facial redness and reduced *Demodex* density (Dd) in patients with high counts of these mites who were suffering from rosacea [46]. Different single-blinded, randomized split-face studies have found that both topical ivermectin and metronidazole effectively treat persistent erythema, even in patients with low or no *Demodex* mites. Ivermectin may provide greater relief from subjective symptoms such as pruritis, burning sensations, and skin roughness. The low *Demodex* counts in some study cohorts could explain the lack of statistical difference between the two treatments, as their anti-parasitic effects are not significantly different. Further research is needed to explore the anti-inflammatory effects of these agents in rosacea patients, particularly those with low or absent *Demodex* levels [47]. Florencia Valvecchia et al. have proven that treatment with topical 1% ivermectin ointment, applied once daily for 2 months, is effective in alleviating the symptoms of patients with *Demodex*-induced blepharitis. Daily use for 2 months reduced the number of collarettes and conjunctival redness [48]. Topical ivermectin allows remission of *Demodex* invasion and clinical improvement for a significant period of time. A maintenance therapy with ivermectin applied twice a week has been proposed to limit relapses [45].

Topical acaricides such as permethrin 5%, benzyl benzoate 10–25%, crotamiton 10%, lindane 1%, and malathion 0.5% have been approved for treating scabies, but current evidence regarding their efficacy in treating demodicosis is quite limited [49]. Permethrin has been evaluated in multiple studies, although its frequency of use varied. When applied every other day, there was a mediocre decrease in Dd, along with some skin irritation. In contrast, daily and twice-daily applications led to significant reductions in Dd and notable clinical improvement of papules, respectively. These findings suggest that increased use of permethrin is effective for *Demodex*-associated dermatoses; however, the potential for skin irritation must be considered. Importantly, while permethrin did cause some irritation, it did not lead to the discontinuation of therapy in the studies, indicating that it is generally well tolerated [50]. W. Chen and G. Plewig demonstrated the effectiveness of 10% topical benzyl benzoate in eliminating *Demodex* mites in their work, but only in a limited number of patients [49].

Initially developed for treating parasites such as Trichomonas vaginalis, metronidazole is now widely used for bacterial infections. It induces cell death by inhibiting protein synthesis through DNA interaction. Although topical metronidazole (0.75%) has shown anti-inflammatory effects and reduced Dd, it can also kill these mites, albeit less effectively than ivermectin and essential oils. It is still uncertain whether treating rosacea with oral low-dose tetracycline or macrolide antibiotics, along with topical azelaic acid or metronidazole, primarily exerts an anti-inflammatory effect or whether it also includes a partial acaricidal effect [49]. Stefano Veraldi et al. from the University of Milan described a clinical case involving a 39-year-old female patient with symptoms of acne vulgaris. Microscopic examinations of skin scrapings revealed the presence of *Demodex* spp. (3 mites/cm^2^). Previous treatment with topical clindamycin and tretinoin was ineffective. Only three months of treatment with 0.75% metronidazole cream and oral doxycycline resulted in full remission [51].

Another treatment option is the use of medicinal oils including, among others, tea tree, camphor, bergamot salvia, and peppermint oil [52,53]. Anon Paichitrojjana and colleagues demonstrated in their study that all mites were eliminated within 16 min of exposure to Thai herbal essential oils, particularly lemongrass, sweet basil, and clove oil, which killed them in just 4 min [54]. This indicates a strong in vitro acaricidal effect. Other therapy methods include sulfur ointment and choline esterase inhibitors. Special heating glasses and infrared irradiation may also be used [52].

Topical therapies for treating demodicosis should be used cautiously due to the sensitive skin of patients, as they can cause irritation. In more severe cases or in patients with sensitive skin, oral treatment should be considered. It is essential to maintain treatment until symptoms completely subside and Dd returns to normal, as relapses are frequent [54].

An association was observed between lifestyle and environmental factors (inadequate water consumption, sporadic exercise, limited bowel movements, more frequent pet ownership, alcohol intake, and smoking) and *Demodex*-related skin diseases, including demodicosis, rosacea, and perioral dermatitis. These factors, linked to gut microbiota, may provide valuable insights into the treatment of these skin conditions and suggest promising directions for future research [55].

## 3. Conclusions

In conclusion, the correct diagnosis of demodicosis continues to be a significant clinical problem due to the non-specificity of symptoms. To establish a diagnosis, direct microscopic examination, standardized skin surface biopsy, skin biopsy, or a combination of these methods is required. Effective treatment that leads to the resolution of symptoms can significantly improve patients’ quality of life, which should be the primary goal of every clinician. Dermatologists should recognize that demodicosis is not an uncommon skin condition, and there are still many aspects of it that remain poorly understood, warranting further research.

## Figures and Tables

**Table 1:** Summary of drugs used in treatment of demodicosis.

Drug	Mechanism of Action	Route of Administration	Form	Side Effects
Ivermectin	Binding to glutamate-gated chloride ion channels in the nervous system, which increases chloride ion permeability, leading to paralysis of the peripheral nervous system	Topical, oral	Cream, tablets	Topical and systemic forms: erythema, itching sensation, exacerbation of rosacea (die-off reaction), mild skin irritation
Permethrin 5%	Disturbance of the flow of potassium and sodium ions in the channels regulating the polarization of the neuronal membrane, which results in paralysis of the central and peripheral nervous system	Topical	Cream, gel, shampoo	Erythema, skin irritation
Benzyl benzoate 10–25%	Unknown	Topical	Cream, gel, shampoo	Burning sensation, skin irritation
Crotamiton 10%	Unknown	Topical	Cream, solution	Erythema, skin irritation, contact dermatitis
Lindane 1%	Blockage of GABA-gated chloride channels, leading to hyperexcitation of the central nervous system	Topical	Lotion	Skin irritation, contact dermatitis
Malathion 0.5%	Inhibition of acetylocholinesterase, which results in overstimulaion of the nervous system	Topical	Shampoo	Skin irritation
Metronidazole	Breakdown of DNA structure, leading to the inhibition of protein synthesis	Topical, oral	Cream, gel, emulsion, tablets	Systemic forms: nausea, vomiting, diarrhea, abdominal pain, metallic taste, headache, and others
Tea tree oil	Inhibition of acetylocholinesterase, resulting in hyperexcitation of the nervous system	Topical	Oil	Skin irritation

## Abbreviations

The following abbreviations are used in this manuscript:

AV	Acne vulgaris
CD	Cylindrical dandruff
Dd	*Demodex* spp. density
DME	Direct microscopic examination
ETR	Erythema-telangiectatic rosacea
PCR	Polymerase chain reaction
SD	Seborrheic dermatitis
SSSB	Standardized skin surface biopsy
TLR2	Type 2 Toll-like receptors
